# A framework for motion correction of background suppressed arterial spin labeling perfusion images acquired with simultaneous multi‐slice EPI

**DOI:** 10.1002/mrm.27499

**Published:** 2018-10-12

**Authors:** Yuriko Suzuki, Thomas W. Okell, Michael A. Chappell, Matthias J.P. van Osch

**Affiliations:** ^1^ C.J. Gorter Center for High Field MRI Department of Radiology Leiden University Medical Center Leiden The Netherlands; ^2^ Wellcome Centre for Integrative Neuroimaging FMRIB Nuffield Department of Clinical Neurosciences University of Oxford Oxford United Kingdom; ^3^ Institute of Biomedical Engineering University of Oxford Oxford United Kingdom

**Keywords:** arterial spin labeling (ASL), background suppression, perfusion image, simultaneous multi‐slice (SMS)

## Abstract

**Purpose:**

When using simultaneous multi‐slice (SMS) EPI for background suppressed (BGS) arterial spin labeling (ASL), correction of through‐plane motion could introduce artefacts, because the slices with most effective BGS are adjacent to slices with the least BGS. In this study, a new framework is presented to correct for such artefacts.

**Methods:**

The proposed framework consists of 3 steps: (1) homogenization of the static tissue signal over the different slices to eliminate most inter‐slice differences because of different levels of BGS, (2) application of motion correction, and (3) extraction of a perfusion‐weighted signal using a general linear model. The proposed framework was evaluated by simulations and a functional ASL study with intentional head motion.

**Results:**

Simulation studies demonstrated that the strong signal differences between slices with the most and least effective BGS caused sub‐optimal estimation of motion parameters when through‐plane motion was present. Although use of the M_0_ image as the reference for registration allowed 82% improvement of motion estimation for through‐plane motion, it still led to residual subtraction errors caused by different static tissue signal between control and label because of different BGS levels. By using our proposed framework, those problems were minimized, and the accuracy of CBF estimation was improved. Moreover, the functional ASL study showed improved detection of visual and motor activation when applying the framework as compared to conventional motion correction, as well as when motion correction was completely omitted.

**Conclusion:**

When combining BGS‐ASL with SMS‐EPI, particular attention is needed to avoid artefacts introduced by motion correction. With the proposed framework, these issues are minimized.

## 1 INTRODUCTION

Simultaneous multi‐slice (SMS, a.k.a multiband) EPI excites multiple slices at the same time and therefore reduces the number of excitation pulses per TR.^1^, ^2^ This approach has been proven to be very advantageous for FMRI and DTI.^1^, ^3^ Whereas for FMRI and DTI, the prime advantage of SMS‐EPI is the acceleration of the acquisition by shortening TR, for arterial spin labeling (ASL) this is much less beneficial because the preparation module for labeling and post‐labeling delay (PLD) is the main time‐consuming part of the sequence and not the readout. For measurement of tracer kinetics using multi time‐point ASL, however, SMS allows the number of slices to be increased within a limited acquisition window to achieve whole‐brain coverage.^4^, ^5^ Another advantage of SMS for 2D‐multislice‐ASL is smaller variation of the level of background suppression (BGS)^6^ and PLD^7^ over the acquired slices. Most ASL sequences now use BGS to decrease physiological noise and motion artefacts from background static tissue, thereby improving the SNR. BGS is highly effective in 3D multi‐shot readout sequences because the image data is acquired after a single excitation per TR, which can be timed to have optimal BGS.^8^ However, for certain applications such as ASL‐FMRI, a single‐shot readout is preferred to achieve high temporal resolution, hence multi‐slice single‐shot EPI is still common as a readout module for ASL.^9^ In multi‐slice ASL, optimal BGS is usually timed to occur for the first slice, whereas longitudinal relaxation will reduce the effectiveness of BGS for more distal slices that are typically acquired hundreds of milliseconds later than the first slice. Similarly, the effective PLD of the distal slices will be hundreds of milliseconds longer than the PLD of the first slice, leading to interpretation issues as well as a loss of SNR in more distal slices. Therefore, shortening the total readout duration by use of SMS‐EPI could help to minimize both detrimental effects.

**Figure 1:**
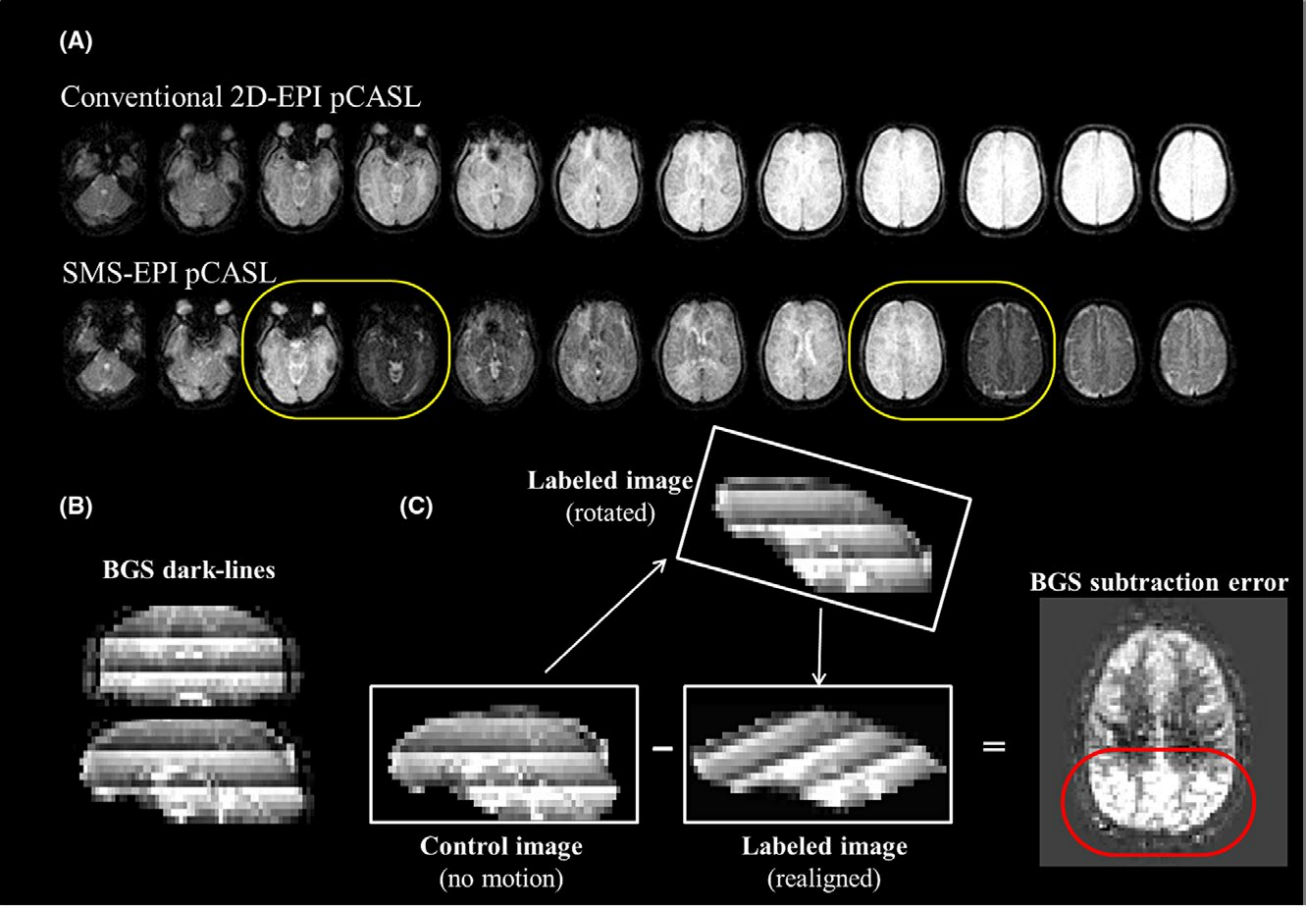
Schematic figures illustrating the potential issues that could arise when motion correction (MoCo) is applied to background suppressed (BGS) ASL acquired with simultaneous multi‐slice (SMS) EPI. A, BGS image of conventional 2D‐EPI and SMS‐EPI pCASL, where yellow circles show slices with the most effective and poorest BGS immediately next to each other. (B) Dark lines observed on reformatted images, produced by slices with the most effective and poorest BGS (BGS dark lines). (C) Subtraction between a control image without motion and a label image with through‐plane rotation (realigned). The red circle shows the subtraction errors caused by the different background static signal intensity between control and label images (BGS subtraction errors)

The combination of SMS and BGS, although desirable for the reasons outlined above, could also potentially introduce new problems when motion correction (MoCo) is required as illustrated in Figure 1. In Figure 1, it can be seen that, with the SMS‐excitation, slices with the most effective BGS will be adjacent to slices that experience the least effective BGS (see the 2 slices indicated with yellow circles in Figure 1A), which results in discrete dark lines clearly visible on sagittal and coronal reformatted images as shown in Figure 1B. These will be referred to as “BGS dark lines” throughout this article. The presence of the BGS dark lines might hamper a successful registration procedure that is required for good MoCo, because such a registration algorithm might attempt to align the BGS dark lines, instead of registering the underlying anatomic structures. Therefore, the motion could be poorly estimated, especially when motion occurs in the slice‐selection direction as a result of rotation around the x‐ or y‐axes or translation in the z‐direction. Furthermore, and more importantly, even assuming registration works correctly, another problem will occur: when a certain part of the cortex moves in the slice direction during either the label or control condition (e.g., a control image without motion and a labeled image with motion, as illustrated in Figure 1C), the tissue would experience different levels of BGS for the label and control conditions. The difference of BGS effectiveness will be especially significant when tissue moves between slice locations with the most and least effective BGS. In such a situation, even when the registration worked successfully and the tissue is correctly realigned to its original location, severe subtraction errors would occur (red circle in Figure 1C) as a result of the significant difference in static tissue signal for the label and control conditions. This problem will be referred to as “BGS subtraction error” throughout this article.

We present a new framework to deal with both sources of error in BGS‐SMS‐ASL imaging, which will enable accurate MoCo realignment as well as a separation of the perfusion‐weighted signal from the above‐mentioned BGS subtraction errors.

## 2 METHODS

### 2.1 Correction framework

The proposed framework consists of 3 steps.

First, homogenization of static tissue signal over the different slices is performed to reduce inter‐slice signal intensity differences as caused by the SMS acquisition with BGS. The slice‐wise mean BGS effect is estimated by

(1) $${\mathrm{BGS}}_{\mathrm{effect}}\left(slice\;z\right)=\;\frac{mean\;tissue\;value\;of\;slice\;z\;of\;M<sub>0</sub>\;image}{mean\;tissue\;value\;of\;slice\;z\;of\;the\;BGS-ASL\;image},$$

where the averaging for the mean tissue value of BGS‐ASL was performed over all dynamic volumes, and homogenization is performed by multiplying the BGS‐ASL time series images by BGS_effect_. Although the main purpose of homogenization is to minimize BGS subtraction errors by homogenizing the inter‐slice signal intensity, it will also avoid erroneous MoCo estimation because of the BGS dark lines as shown in Figure 1B.

Second, MoCo registration based on the conventional rigid‐body transformation is performed by commonly available software such as SPM^10^ or FSL,^11^ while using the M_0_ image as reference, which is chosen based on the absence of dark lines. In addition, an extra 4D‐data set of the same size is created, which at first has a constant value equal to BGS_effect_ for each slice, but is subsequently resliced according to the motion parameters as estimated by the MoCo for the ASL time series. This new data set will be referred to as “resliced BGS_effect_” and used in the third step.

Third, perfusion‐weighted signal is extracted. A standard subtraction of label from control images would not extract the perfusion‐weighted signal correctly, because the perfusion‐weighted signal was scaled by the BGS_effect_ during the homogenization process of the first step. Moreover, because the application of BGS_effect_ only achieves slicewise homogenization of the static tissue signal, there will remain some BGS‐variations at the voxel‐level that will still lead to some residual BGS subtraction errors when through‐slice motion occurs. To correct for both effects, it is proposed to use a general linear model (GLM) regression to (1) rescale the perfusion‐weighted signal as well as to (2) separate the perfusion‐weighted signal from residual errors. The GLM is represented as

(2) $$y_{t}=\;\beta_{\mathrm{baseline}}x_{t\_baseline}+\;\beta_{\mathrm{perf}}x_{t\_perf}+\beta_{\mathrm{error}}x_{t\_error}+\;e,$$

where *y_t_* is the motion corrected ASL time series at a certain voxel. The regressors that constitute the design matrix are: *x_t_baseline _*= 1 for baseline tissue signal, *x_t_perf_* that describes the labeling paradigm [0.5, −0.5, … , 0.5, −0.5]^T^ and *x_t_error_*that represents an estimate of the pixel‐wise residual error. As mentioned above, because the perfusion‐weighted signal was scaled by the BGS_effect_ during the homogenization process, *x_t_perf_* was also multiplied by the resliced BGS_effect_ that was generated at the second step. It should be noted that the resliced BGS_effect_ will both depend on the location within the images as well as on the temporal profile and extent of the through plane motion. In this study, *x_t_error_*was generated from subtraction of the homogenized (BGS‐removed) ASL time series before and after MoCo. This regressor will be dominated by static tissue signal changes and will therefore be a reasonable surrogate for the residual error after MoCo. β_baseline_, β_perf_, and β_error_ are fitting coefficients for *x_t_baseline_*, *x_t_perf_*, and *x_t_error_* i.e., β_baseline_ and β_perf_ represent the homogenized background tissue image and the baseline perfusion‐weighted (∆M) image, respectively. *e* is the fitting error. When ASL‐FMRI is performed, an additional regressor is added

(3) $$y_{t}=\;\beta_{\mathrm{baseline}}x_{t\_baseline}+\;\beta_{\mathrm{perf}}x_{t\_perf}+\beta_{act\_perf}x_{t\_act\_perf}+\;\beta_{\mathrm{error}}x_{t\_error}+\;e,$$

where x_t_act_perf_ describes the perfusion signal changes as a result of activation and β_act_perf_ is the fitting coefficients for x_t_act_perf_. Please note that x_t_act_perf_ is also multiplied by the resliced BGS_effect_, similar as described above.

### 2.2 Simulation

All simulations and image processing were performed offline using SPM 12 and custom‐written scripts in MATLAB (The MathWorks, Natick, MA).

A data set of single‐PLD pseudo‐continuous ASL (pCASL), M_0_ image, and a quantitative T_1_ map, all at the same spatial resolution, were used from a single subject out of a previous in‐vivo healthy volunteer perfusion study (see Table 1 in Heijtel et al.^12^ for acquisition details). The quantitative CBF map was calculated in accordance with the recommendation from the recent ASL white paper^8^

(4) $$CBF=\frac{6000∙\lambda ∙\Delta M∙exp(\frac{\mathrm{PLD}}{T_{1,blood}})∙}{2∙\alpha ∙T_{1,blood}∙M_{0}∙\left(1-exp\left(-\frac{\tau }{T_{1,blood}}\right)\right)}\;,$$

where ∆M is the perfusion‐weighted signal intensity, M_0_ is the signal intensity of the M_0_‐scan, and τ is the labeling duration. The values from the white paper for the brain–blood partition coefficient (λ) of 0.95 mL/g, the T_1,blood_ of 1650 ms, and labeling efficiency (α) of 0.85 were used.

**Figure 2:**
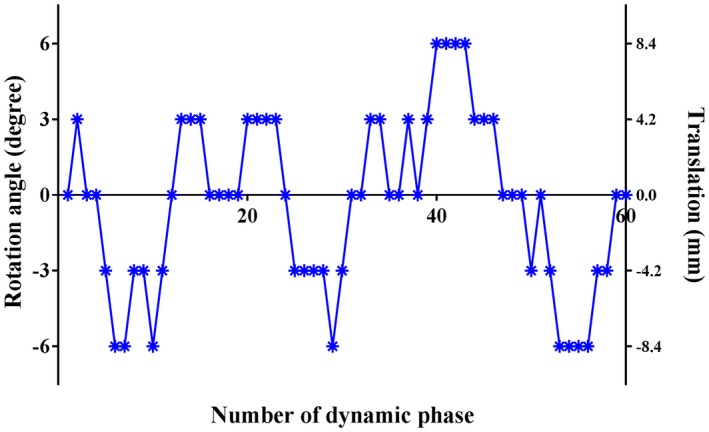
Motion applied to the simulated data set: ±1.4 and 2.8 fractional pixel translation (±4.2 and 8.4 mm) in the x‐ and y‐direction, ±0.6 and 1.2 fractional slice shift (±4.2 and 8.4 mm) in z‐direction, and ±3 and 6° rotation around the x‐, y‐, and z‐axes

From the M_0_ images, 7 time series of 60 dynamic images each were generated, and 6 different types of motion (translation in the x‐, y‐, and z‐direction and rotation around the x‐, y‐ and z‐axes) were applied to 6 of them. As illustrated in Figure 2, each type of motion has a 4‐step pattern in a single time series: ±1.4 and ±2.8 fractional pixel translation (±4.2 and ±8.4 mm) in the x‐ and y‐direction, ±0.6 and ±1.2 fractional slice shift (±4.2 and ±8.4 mm) in the z‐direction, and ±3 and ±6° rotation around the x‐, y‐, and z‐axes. One time series was untouched (no‐motion data set). By using the T_1_ map, the pixel‐wise signal attenuation as a result of 2 BGS inversion pulses (TI = 1860 and 3150 ms) was calculated, in which different PLDs for the multi‐slice acquisition were incorporated by assuming an interval of 30 ms between 2 subsequent excitation pulses; SMS acquisition with factor 3 was assumed and the following standard Bloch‐equation was used for calculating the evolution of the longitudinal magnetization

(5) $$M_{z}\left(t\right)=M_{0}\left(1-exp\left(-\frac{t}{T_{1}}\right)\right)+M_{z}\left(0\right)exp\left(-\frac{t}{T_{1}}\right),$$

where M_z_(t) is the longitudinal magnetization at time point t, M_0_ and T_1_ are pixel‐wise values from the M_0_ image and T_1_ map, respectively. Afterward, perfusion‐like signal changes were incorporated to the even dynamic numbers to represent the labeled images. Perfusion‐like signal was only added to the left side of the brain, thereby keeping the right side without perfusion, in which all signal intensity that would appear after the post‐processing will be a measure of the artefactual signal as a result of applied post‐processing without any influence of the underlying perfusion pattern. Using these data sets, 2 studies were carried out.

#### 2.2.1 Simulation 1: comparison of MoCo estimation

First, we studied whether MoCo would indeed be impaired in SMS‐ASL data with BGS that exhibit the BGS dark lines and whether the performance of MoCo would be improved by the homogenization step (first step of the correction framework) as well as by using the M_0_ image as reference for registration, instead of 1 dynamic out of the SMS‐ASL data set. All 6 motion‐corrupted data sets underwent 4 types of MoCo registration defined as follows:

*MoCo‐A*: with BGS dark lines present and the use of the firstly acquired ASL image as reference
*MoCo‐B*: with BGS dark lines present and the use of the M_0_ image as reference (instead of the firstly acquired ASL image)
*MoCo‐C*: with homogenization and the use of the firstly acquired ASL image as reference
*MoCo‐D*: with homogenization and the use of the M_0_ image as reference

As a reference for the MoCo performance comparison, another set of 6 motion‐corrupted data sets was created with exactly the same motion patterns, but without BGS‐ and perfusion‐like signal attenuation, that also underwent the MoCo registration (referred to as “ref‐no‐BGS”). This separate reference data set allows any errors resulting from the suboptimal performance of MoCo unrelated to BGS dark lines to be excluded from the evaluations. For each 6 time series with different types of motion as described above, 6 motion parameters (translation in x‐, y‐, and z‐direction and rotation around x‐, y‐, and z‐direction) were estimated by *MoCo‐A*, *‐B*, *‐C*, and *‐D*, and the normalized mean difference with regard to the reference was calculated, which was normalized by the size of the simulated translation and rotation.

#### 2.2.2 Simulation 2: separation of the perfusion‐weighted signal

All 6 motion‐corrupted data sets underwent the proposed post‐processing framework (i.e., the homogenization, MoCo [*MoCo‐D* in simulation‐1], and GLM as described above) that will be referred to as “*NewMoCo.*” Using the obtained β_perf_ map (i.e., ∆M image) and the M_0_ image, the CBF map was calculated by applying Equation (4). For comparison, CBF maps were also calculated using ∆M images obtained by GLM but without the homogenization step (*MoCo‐B* in simulation 1), referred to as “*StdMoCo*,” GLM without both the homogenization step and MoCo, referred to as “*NoMoCo*,” and also with the same framework as *NewMoCo*, but without including the error regressor “*x_t_error_*” in the design matrix, referred to as “*NewMoCo_wo_error_reg_*.”

All signal intensities observed in the right side of the brain represent the level of artefactual signal arising from the applied post‐processing, i.e., the signal intensity will be close to zero when errors such as BGS subtraction errors as well as conventional type of subtraction error because of inaccurate motion estimation (or when MoCo is not applied) are corrected well.

### 2.3 In vivo ASL‐FMRI study with healthy volunteers

The study was approved by the local institutional review board and all volunteers provided written informed consent before inclusion into this study. A total of 4 volunteers (2 male, 2 female, mean age = 41.8 y [range, 24–59 y]) without known cerebrovascular disease participated in the study.

Three ASL‐FMRI scans using pCASL were performed for each volunteer consisting of a blue‐and‐yellow 8 Hz flickering circular checkerboard (for visual stimuli) for 32 s alternated with a white fixation cross on a black background for 32 s. Volunteers were instructed to also perform a bilateral finger tapping task while the checkerboard was projected. For the first and the last ASL‐FMRI scans, volunteers were instructed to move their head during pCASL labeling and/or PLD (not during the readout). For the second ASL‐FMRI scan, volunteers were instructed not to move, and this scan was used as a reference. After the first ASL‐FMRI scan, image data were immediately processed to estimate the motion so that before the third ASL‐FMRI scan, the volunteers could be instructed to adjust their degree of motion; we aimed for a few mm and/or degrees of motion. The M_0_ scan was acquired between the first and second ASL‐FMRI scans.

All MR scans were performed on a Philips 3.0T Ingenia scanner (Philips, Best, The Netherlands) using a 32‐channel head coil. Imaging parameters for SMS‐EPI pCASL were as follows: FOV = 240 × 240 mm, scan matrix = 80 × 80, 18 slices acquired with a thickness of 7.0 mm, TE of 16 ms and TR of 4000 ms, sensitivity‐encoding (SENSE) was applied with a factor of 3 in anterior‐posterior direction, and the SMS‐factor was set to 3. Both labeling duration and PLD were set to 1800 ms. A fat‐suppression pre‐pulse was applied to avoid water–fat shift artifacts and a WET pre‐saturation scheme was inserted before labeling^13^, ^14^; BGS pulses were applied using hyperbolic secant pulses^15^ at 1830 and 3150 ms after the start of labeling. The timing of the BGS pulses was determined via Bloch‐simulations. With the number of dynamic scans set to 64, the total scan time was 8 min 40 s. The M_0_ image was acquired without labeling, WET pre‐saturation and BGS but with identical acquisition parameters as the perfusion scan except for the TR, which was set to 2500 ms. Before the quantification process, the M_0_ image was multiplied by the factor 1/(1 − exp(−TR/T_1_gm_)), where T_1_gm_ was assumed to be 1200 ms, to correct for incomplete T_1_ recovery.^8^, ^16^

**Figure 3:**
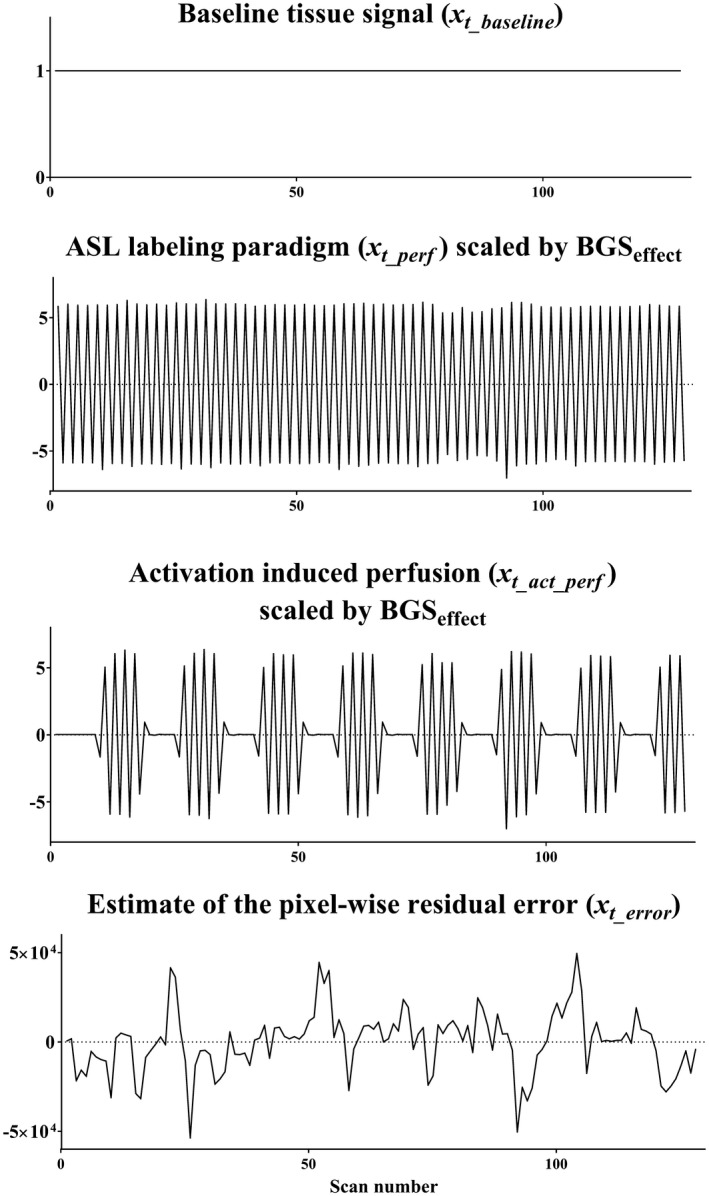
An example of the design matrix used to the in vivo ASL‐FMRI study, in which *x_t_baseline _*= 1 for baseline tissue signal, *x_t_perf_* represents the signal changes as a result of the ASL labeling paradigm, and *x_t_act_perf_* describes the ASL signal changes induced by activation. Because the perfusion‐weighted signal is scaled by the BGS_effect_ during the homogenization process, *x_t_perf_* and *x_t_act_perf_* were multiplied by the resliced BGS_effect_ that was generated at the second step to include the pixel‐by‐pixel scaling of the perfusion‐weighted signal. *x_t_error_*represents an estimate of the pixel‐wise residual error

All 3 ASL‐FMRI data sets were post‐processed with the *NewMoCo* framework, and the baseline CBF map was calculated from the β_perf_ map. Moreover, a map that consisted of the t‐value of β_act_perf_ was generated to indicate the area where the perfusion changed as a result of the visual and motor tasks. Figure 3 shows an example of the used design matrix. For the 2 ASL‐FMRI data sets with intentional head movement, *NoMoCo* and *StdMoCo* were also performed for comparison.

On the t‐value maps obtained from the ASL‐FMRI data set without intentional head movement, ROIs were manually drawn around the activated visual, and left and right motor area. These ROIs were copied to the t‐value maps of the data sets with intentional head movement, and the mean t‐values as well as the number of voxels that exhibited a t‐value >3.0 were compared between *NewMoCo*, *NoMoCo*, and *StdMoCo*. The analysis was performed by a 2‐way ANOVA followed by multiple comparisons with adjustment of Bonferroni using SPSS.

## 3 RESULTS

### 3.1 Simulation 1: comparison of MoCo estimation

**Figure 4:**
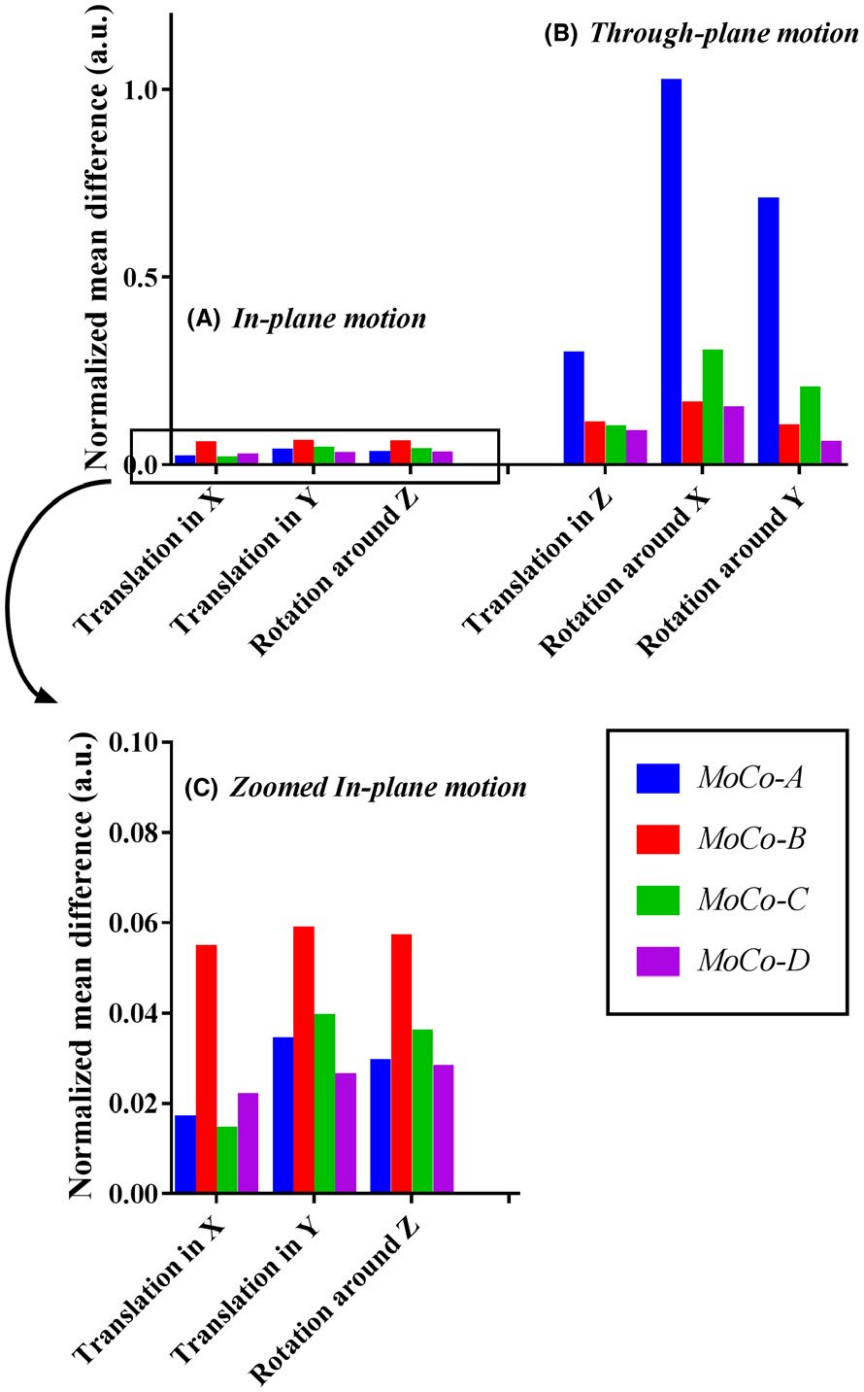
Normalized mean difference of MoCo estimation for (A) in‐plane motion and (B) through‐plane motion obtained from simulation 1. (C) The result of in‐plane motion shown in (A) is given in more detail. MoCo‐A, with BGS dark lines present and the use of the firstly acquired ASL‐image as reference; MoCo‐B, with BGS dark lines present and the use of the M0 image as reference (instead of the firstly acquired ASL‐image); MoCo‐C, with homogenization and the use of the firstly acquired ASL‐image as reference; MoCo‐D, with homogenization and the use of the M0 image as reference.

Figure 4 shows the normalized mean difference of MoCo estimated motion parameters with *MoCo‐A*, *‐B*, *‐C*, and *‐D *relative to the reference. For correction of through‐plane motion (Figure 4B) *MoCo‐A* resulted in the largest mean normalized difference from the reference, which can be attributed to the presence of BGS dark lines influencing the MoCo estimation. When using the M_0_ image as a reference (*MoCo‐B*) and homogenization (*MoCo‐C*), as well as the combination of these two (*MoCo‐D*), the mean normalized difference was much lower. In contrast, for correction of in‐plane motion (Figure 4A) differences of MoCo estimation were much smaller for *MoCo‐A*, presumably because the presence of BGS dark lines did not influence the MoCo estimation for in‐plane motion. When observing in more detail these results as in the zoomed version of Figure 4C, it becomes clear that *MoCo‐B* showed relatively larger mean normalized difference for in‐plane motion than the other approaches. It can be speculated that the use of the M_0_ image as a reference for registration might introduce small additional errors because of its slightly different contrast as compared to pCASL images.

For further simulations (simulation 2, see next section) and the in vivo study, *MoCo‐B* and *MoCo‐D* were chosen and will be referred to as *StdMoCo* and *NewMoCo*, respectively.

### 3.2 Simulation 2: separation of perfusion‐weighted signal

**Figure 5:**
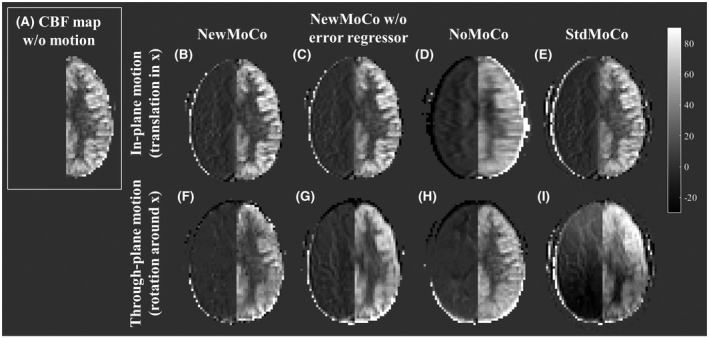
Representative CBF maps obtained from simulation 2. In the right hemisphere, no perfusion signal was simulated, and therefore all signal intensity in the right hemisphere indicates motion‐related errors. (A) Reference CBF map obtained from the data set without motion. (B–E) Respectively *NewMoCo*, *NewMoCo* without error regressor, *NoMoCo*, and *StdMoCo* are applied to in‐plane motion (translation in the x‐direction) and (F–I) to through‐plane motion (rotation around the x‐axis)

Figure 5 illustrates the simulation results from the slice that exhibited the largest difference in static tissue signal intensity compared to a neighboring slice and therefore most prone to the BGS subtraction errors. In *StdMoCo*, severe BGS subtraction errors were observed for through‐plane motion (severe signal increase in the anterior part of the brain in combination with a decrease in signal at the posterior side, as illustrated in Figure 5I), whereas such BGS subtraction errors were not observed in images with in‐plane motion (Figure 5E). By use of the *NewMoCo *approach, the BGS subtraction errors were substantially reduced for through‐plane motion (Figure 5F). When using *NoMoCo*, the presence of in‐plane motion appeared as a blurring of the CBF map (Figure 5D). In contrast, for through‐plane motion (Figure 5H) such a blurring was less obvious. In both types of motion, however, the conventional subtraction errors (because of motion) were observed clearly along tissue boundaries of the right side of the brain (without perfusion). When removing the error regressor “*x_t_error_*” from the design matrix (*NewMoCo_wo_error_reg_*), a small increase of blurring was observed in the image with through‐plane motion, especially along the boundary between gray and white matter (Figure 5G).

**Figure 6:**
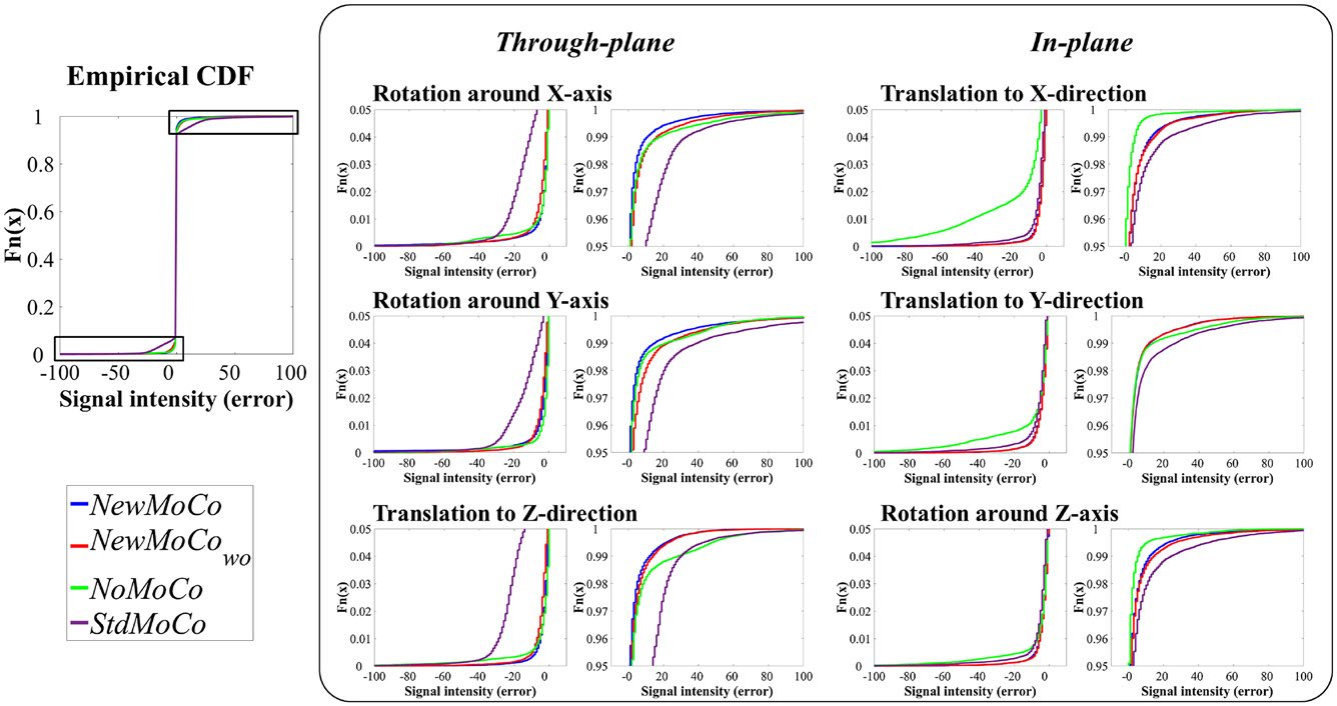
The empirical cumulative distribution function (CDF) of the signal intensity in the right side of the brain (without perfusion) obtained from simulation 2, which indicates the level of artefactual signal that arises mostly from conventional subtraction errors because of motion as well as the above‐mentioned BGS subtraction errors. The value is ideally close to zero when both sources of errors are well corrected. For magnified presentation, only plots with the cumulative distribution from 0–0.05 and 0.95–1.00 and signal intensity between −100 to 100 are shown

For a more quantitative observation of the artefactual signal changes, Figure 6 shows the empirical cumulative distribution function (CDF) of the signal intensity in the right side of the brain (without perfusion), in which without any errors present (i.e., all voxels are zero) the empirical CDF would be a step function at zero, whereas significant deviation from this step‐function at zero implies the presence of voxels with larger errors. (The asymmetry in the empirical CDF between positive and negative signal intensities is attributed to the fact that the signal intensity was analyzed only for the right side of the brain.) Similar to the qualitative results as shown in Figure 5, more pixels with signal intensity far from zero were observed as a result of *StdMoCo* applied to data corrupted by through‐plane motion, whereas the distribution was closer to the step‐function for in‐plane motion. For some motion patterns (not clearly divided by the category “through‐plane” and “in‐plane motion”), *NoMoCo* resulted in mild elevation of artefactual signal, which reflects the blurring as also observed in Figure 5D. With the *NewMoCo *approach, both types of subtraction errors were substantially reduced independent of the direction of motion.

### 3.3 In vivo healthy volunteer study

**Figure 7:**
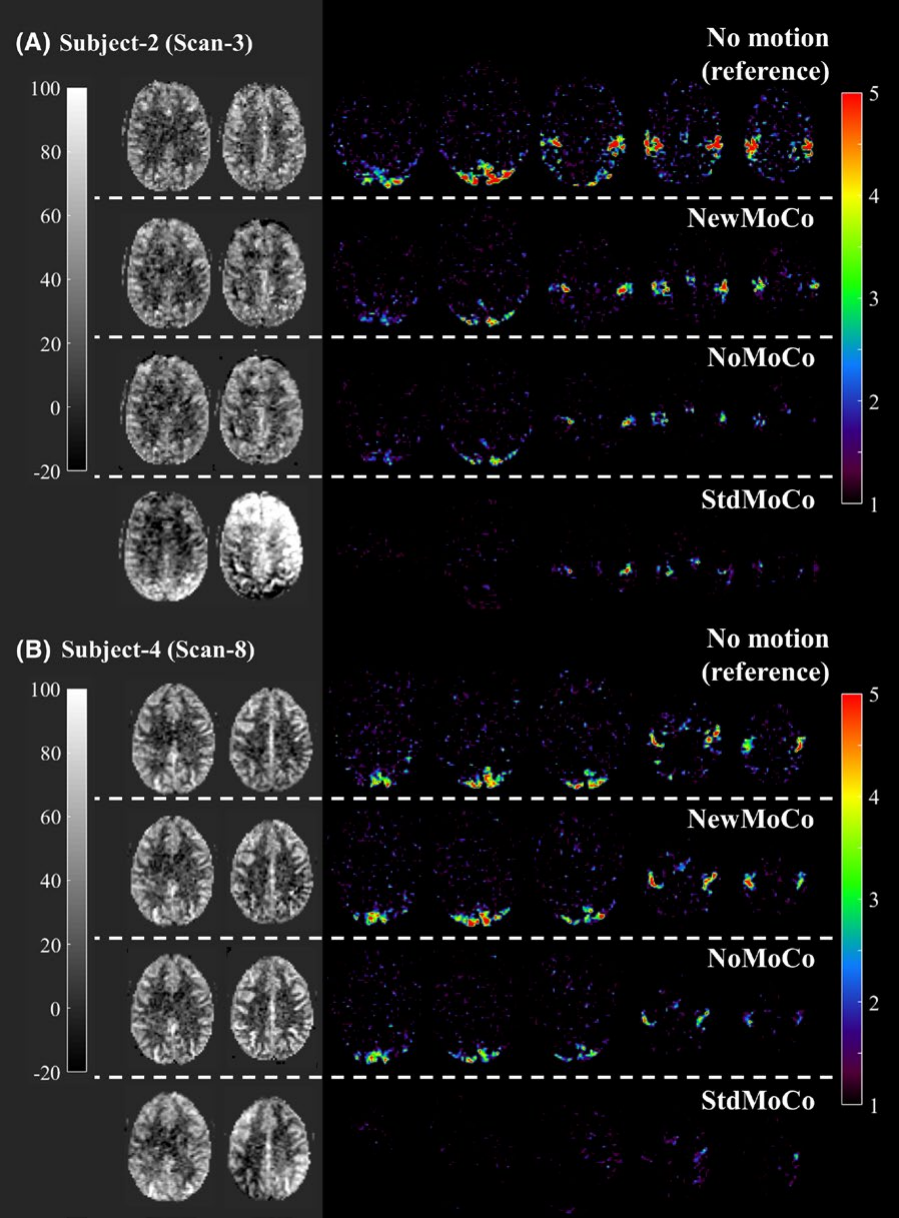
Representative baseline CBF maps and t‐value maps showing the activated regions by the visual stimulus and finger tapping obtained from the ASL‐FMRI study with intentional head movements processed by *NewMoCo*, *NoMoCo*, and *StdMoCo*. As reference, maps acquired without intentional head movement are also shown on top of them

**Figure 8:**
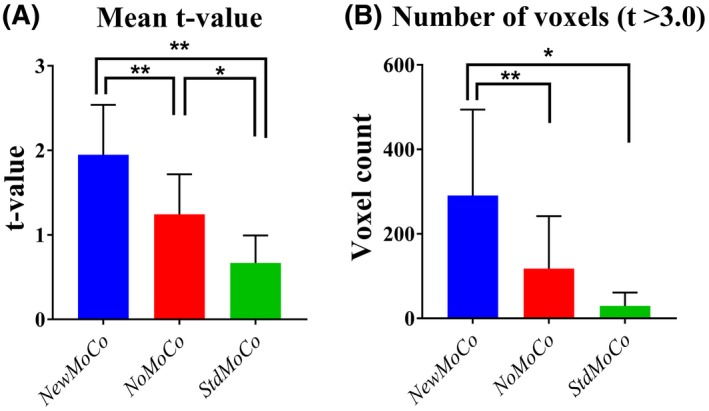
The results of the ROI analysis from all scans. (A) Mean t‐values and (B) number of voxels that exhibited a t‐value >3.0 from data set processed by *NewMoCo*, *NoMoCo*, and *StdMoCo*

Figure 7 shows representative results of the ASL‐FMRI experiments showing the baseline CBF maps as well as the activated regions evoked by the visual stimulus and finger tapping. In the motion‐corrupted data sets, the activated regions were depicted much more clearly when applying *NewMoCo *as compared to *StdMoCo*, whereas *NoMoCo* provided results in between of these 2 approaches. The results of the ROI analysis from all volunteers confirmed that the highest mean t‐value (Figure 8A), as well as highest number of activated voxels, (Figure 8B) were obtained by *NewMoCo*. All differences were statistically significant (*P* < 0.05) except the difference between *NoMoCo* and *StdMoCo* of the number of voxels that exhibited a t‐value >3.0.

**Figure 9:**
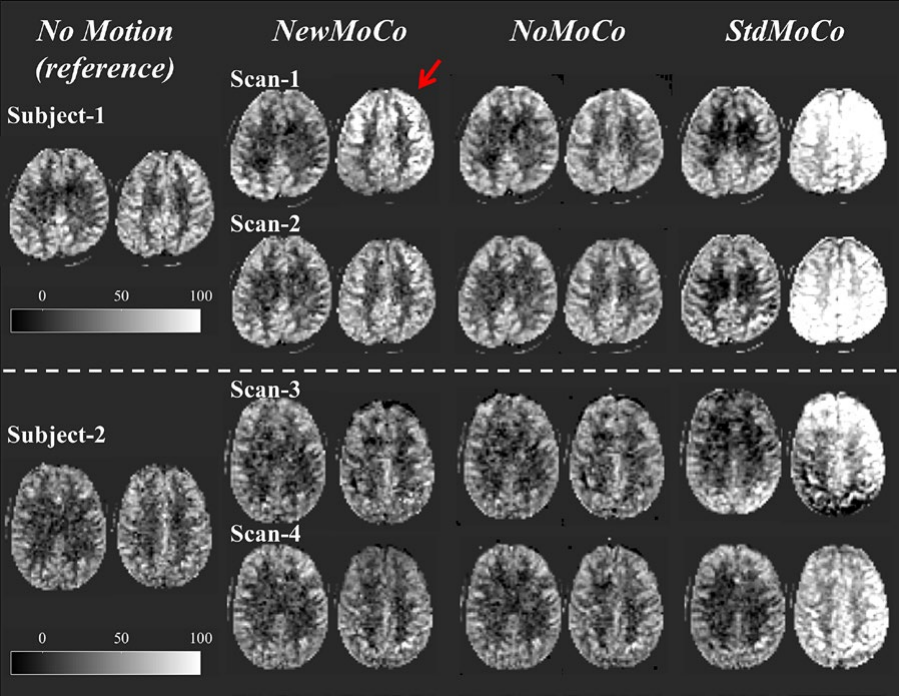
The baseline CBF maps from the subjects 1 and 2 (subjects 3 and 4 are shown in Figure 10) showing 2 slices with largest difference of BGS efficiency from the neighboring slices. Red arrows indicate the residual error from 2 scans with the most severe head‐motion

**Figure 10:**
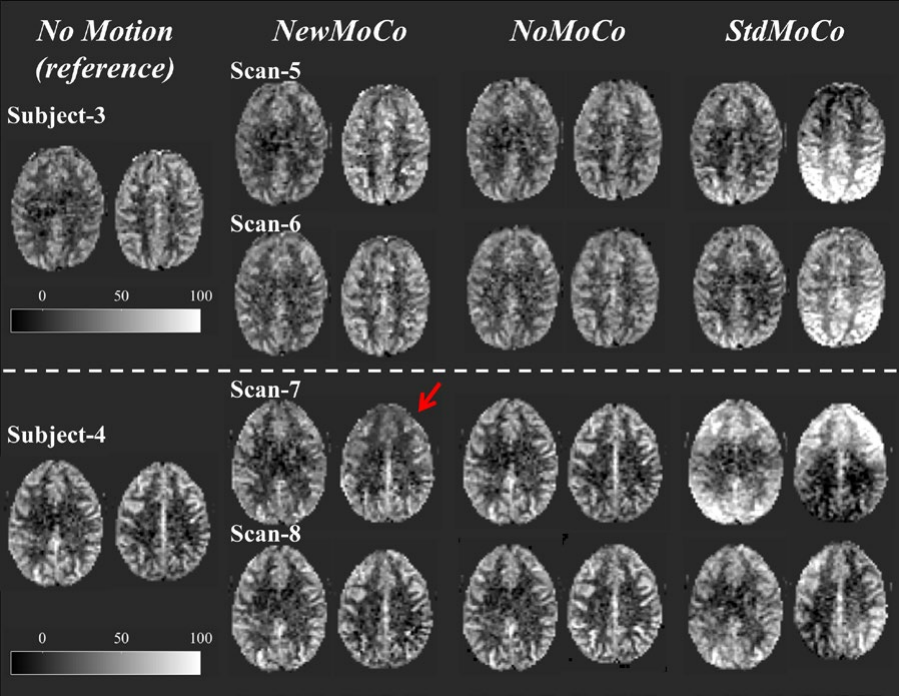
The baseline CBF maps from subjects 3 and 4 (subjects 1 and 2 are shown in Figure 9), showing 2 slices with largest difference of BGS efficiency from the neighboring slices. Red arrows indicate the residual error from 2 scans with the most severe head‐motion

As baseline CBF maps from all scans show (Figures 9, 10), the BGS subtraction errors on the baseline CBF map were corrected well by *NewMoCo*. For the 2 data sets with the most severe head‐motion (scan 7 from volunteer 4 and scan 1 from volunteer 1), however, correction was not sufficient for slices with the largest BGS difference between neighboring slices, as indicated by red arrows in Figures 9, 10. For the baseline CBF maps, *NoMoCo* generally resulted in reasonable image quality without BGS subtraction errors, although some blurring can be observed. The estimated motion parameters from all volunteers are shown in Supporting Information Figure S1.

## 4 DISCUSSION

In this article, we demonstrated 2 major issues that can affect ASL measurements when using a 2D multi‐slice readout with SMS and BGS: sub‐optimal estimation of motion parameters by MoCo and the occurrence of BGS subtraction errors, which can be very severe. Our simulation studies showed that these problems predominantly occur when through‐plane motion (translation in z‐direction or rotation around the x‐ or y‐axes) is present, pointing to the large influence of the BGS dark lines. Although MoCo estimation was already improved to a great extent by the use of the M_0_ image as the reference for MoCo registration, successful realignment still led to BGS subtraction errors in the perfusion‐weighted signal, as was also evident in the ASL‐FMRI study. By use of our proposed framework, the BGS subtraction errors were minimized and the depiction of activated regions was improved, while still allowing the use of conventional MoCo‐methodology as available in commonly applied neuroscience software. The benefit of our framework was most obvious in the functional ASL experiments, in which application of MoCo is especially important to detect statistically significant activation from small regions of interest.

In general, MoCo methods such as those implemented in SPM and FSL estimate motion parameters based on an error‐measure calculated from the similarity between images. Therefore, strong contrast as imposed by the BGS dark lines could overwhelm differences as a result of anatomic misalignment and cause an erroneous estimation of motion parameters. By using the M_0_ image, which does not exhibit BGS dark lines, as the reference image for MoCo estimation, the MoCo will not be biased because of a tendency to keep BGS dark lines aligned and will therefore be better able to pick up the real motion and that will therefore improve the MoCo estimation. This was found to be true for through plane motion, both with and without homogenization. However, for in‐plane motion, the use of M_0_ image as a reference resulted in slightly poorer performance when homogenization had not been applied, although to an extent that was much smaller than the improvement for through‐plane motion. The poorer performance of the M_0_‐approach (*MoCo‐B*) for in‐plane motion can most probably be attributed to the difference in contrast between the M_0_ and BGS‐pCASL images. The improvement observed by combining the M_0_‐approach with the homogenization (*MoCo‐D*) reinforces this argument, in which further reduction of the normalized mean difference was achieved for all types of motion.

The source of subtraction errors as identified in this study can be categorized into 2 groups: the first type of errors are conventional subtraction errors occurring when anatomical structures are not stable at the same location as a result of inaccurate motion estimation because of BGS dark lines (or when MoCo is not applied), i.e., the “normal” type of motion artefacts. One important target of BGS is to minimize this type of subtraction error by lowering the signal intensity of static tissue.^8^ In multi‐slice ASL, however, even when SMS is applied, multiple excitation pulses will still be needed and the level of BGS will not be optimal in all (sets of) slices, thereby increasing the severity of these subtraction errors. In our experiments such artefacts can be observed in the *NoMoCo* data (i.e., subtraction without applying motion correction).

The second type of error arises from subtraction of signals with different levels of BGS (BGS subtraction error). These errors are evident in the *StdMoCo* data in which MoCo does assure that anatomic structures are realigned, although signal intensity differences of static tissue result in artefacts because of the fact that an anatomic structure was part of a different slice during the label versus the control condition (e.g., a certain anatomic structure moves from a location with optimal BGS for the control acquisition to a position with lower BGS‐efficiency during the label condition). We propose to eliminate this second type of subtraction errors by homogenization of the static tissue signal over slices before MoCo is performed. This homogenization is, however, only performed by equalizing the mean signal of a slice relative to the mean signal of the same slice of the M_0_ image. This implies that at the level of individual pixels this homogenization will not be perfect and residual BGS subtraction errors can still occur. To minimize such residual errors, the use of a GLM was proposed with additional regressors, besides a subtraction‐regressor that extracts the perfusion‐weighted signal from the unsubtracted ASL data. In this study, we used the subtracted pixel value of the homogenized ASL data before and after MoCo as an additional regressor. This metric is a measure of static tissue signal change after MoCo reslicing, which is the main cause of BGS subtraction errors. In the simulation study, the calculated CBF map showed increased errors along the boundary between gray and white matter when applying *NewMoCo* without this error regressor, which most likely represents residual BGS subtraction errors.

It should be noted that, as Figures 9, 10 shows, when the motion was very large *NewMoCo* could fail to generate reliable baseline CBF maps at the slices with the largest difference in BGS between neighboring slices (at the BGS dark lines), and *NoMoCo* resulted in better quality. When ASL is performed for clinical diagnostic purposes, the main goal will be to achieve high quality resting‐state baseline CBF map, in which some motion‐blurring can be tolerated. Therefore, performing both *NewMoCo* and *NoMoCo* would be recommended to avoid potential misinterpretation because of residual BGS subtraction errors on the CBF map as obtained by *NewMoCo*. However, when functional ASL is performed, small location changes might result in one becoming blind for the brain activation, thereby making MoCo more essential. In fact, the ROI analysis performed on the ASL‐FMRI scans resulted in higher mean t‐value and higher number of activated voxels by *NewMoCo* than *NoMoCo* for all 8 scans.

Another approach to avoid BGS subtraction errors would be to limit the inter‐slice BGS signal differences by changing the acquisition. Such an approach was proposed recently by Shao et al.^6^ via a slice‐dependent signal preparation (i.e., pre‐saturation is not performed at a single moment in time for all slices), but slice‐dependent preparation pulses are applied to ensure optimal BGS for each slice.^6^ With this approach, the largest differences in signal intensity of static tissue between neighboring slices can be avoided, thereby lowering the risk for the most severe subtraction artefacts, although not excluding these completely. Moreover, when motion occurs between the saturation module and readout (i.e., including the labeling duration and PLD), the carefully optimized scheme could be affected and inter‐slice signal differences could appear. Therefore, it would be interesting to see whether the currently proposed approach would also improve motion‐corrupted SMS‐ASL data acquired with the slice‐dependent pre‐modulation approach. It could be expected that both approaches would enhance each other’s performance.

Several limitations of the current study should be mentioned. First, the most important message of this article is to demonstrate the issues that could be introduced by combined use of SMS and BGS in an ASL study. Although we used the subtraction of pixel values of the homogenized ASL data before and after MoCo as an additional error regressor, it does not mean that we can assure that this is the most optimal regressor. In fact, as discussed above, there is certainly room for further improvements when severe motion is present, especially for the baseline CBF maps. Second, the proposed method does not correct for other well‐known sources of artefacts in motion corrupted MRI data. For example, distortions because of B_0_ inhomogeneities and residual water–fat shift artifacts (even though fat suppression was applied) are dependent on the head orientation and can therefore change during head motion. Therefore, after MoCo realignment, such artifacts will be repositioned to different locations, again resulting in subtraction errors. In this study, only rigid‐body MoCo was applied, which cannot correct for such artifacts. Moreover, motion that happened during the readout might introduce additional artifacts that cannot be corrected by rigid‐body MoCo. In future work, further investigation of even more appropriate regressors and application of more advanced MoCo approaches should be studied.

## 5 CONCLUSION

In ASL with combined use of SMS and BGS, severe BGS subtraction errors can occur when through‐plane motion is corrected by traditional MoCo procedures. With the proposed framework, these BGS subtraction errors can be minimized, resulting in improved accuracy of CBF‐estimation while still allowing the use of conventional MoCo approaches as available in widely used software packages.

## Supporting information


**FIGURE S1** Estimated motion parameters of all scans

## References

[1] Moeller S , Yacoub E , Olman CA , et al. Multiband multislice GE‐EPI at 7 tesla, with 16‐fold acceleration using partial parallel imaging with application to high spatial and temporal whole‐brain FMRI. Magn Reson Med. 2010;63:1144–1153. 20432285 10.1002/mrm.22361 PMC2906244

[2] Setsompop K , Gagoski BA , Polimeni JR , Witzel T , Wedeen VJ , Wald LL . Blipped‐controlled aliasing in parallel imaging for simultaneous multislice echo planar imaging with reduced g‐factor penalty. Magn Reson Med. 2012;67:1210–1224. 21858868 10.1002/mrm.23097 PMC3323676

[3] Feinberg DA , Moeller S , Smith SM , et al. Multiplexed echo planar imaging for sub‐second whole brain FMRI and fast diffusion imaging. PLoS One. 2010;5:e15710. 21187930 10.1371/journal.pone.0015710 PMC3004955

[4] Zhang K , Yun SD , Shah NJ . Tripled readout slices in multi time‐point pCASL using multiband Look‐Locker EPI. PLoS One. 2015;10:e0141108. 26544715 10.1371/journal.pone.0141108 PMC4636240

[5] Feinberg DA , Beckett A , Chen L . Arterial spin labeling with simultaneous multi‐slice echo planar imaging. Magn Reson Med. 2013;70:1500–1506. 24130105 10.1002/mrm.24994 PMC4162886

[6] Shao X , Wang Y , Moeller S , Wang DJ . A constrained slice‐dependent background suppression scheme for simultaneous multislice pseudo‐continuous arterial spin labeling. Magn Reson Med. 2018;79:394–400. 28198576 10.1002/mrm.26643 PMC5557712

[7] Kim T , Shin W , Zhao T , Beall EB , Lowe MJ , Bae KT . Whole brain perfusion measurements using arterial spin labeling with multiband acquisition. Magn Reson Med. 2013;70:1653–1661. 23878098 10.1002/mrm.24880

[8] Alsop DC , Detre JA , Golay X , et al. Recommended implementation of arterial spin‐labeled perfusion MRI for clinical applications: a consensus of the ISMRM perfusion study group and the European consortium for ASL in dementia. Magn Reson Med. 2015;73:102–116. 24715426 10.1002/mrm.25197 PMC4190138

[9] Ivanov D , Poser BA , Huber L , Pfeuffer J , Uludag K . Optimization of simultaneous multislice EPI for concurrent functional perfusion and BOLD signal measurements at 7T. Magn Reson Med. 2017;78:121–129. 27465273 10.1002/mrm.26351 PMC5484334

[10] Ashburner J , Friston K . CHAPTER 4 ‐ rigid body registration In: Penny W, Friston K, Ashburner J, Kiebel S, Nichols T, eds. Statistical parametric mapping: the analysis of functional brain images. London: Academic Press; 2007:49–62.

[11] Jenkinson M , Bannister P , Brady M , Smith S . Improved optimization for the robust and accurate linear registration and motion correction of brain images. Neuroimage. 2002;17:825–841. 12377157 10.1016/s1053-8119(02)91132-8

[12] Heijtel DF , Mutsaerts HJ , Bakker E , et al. Accuracy and precision of pseudo‐continuous arterial spin labeling perfusion during baseline and hypercapnia: a head‐to‐head comparison with ^15^O H_2_O positron emission tomography. Neuroimage. 2014;92:182–192. 24531046 10.1016/j.neuroimage.2014.02.011

[13] Ogg RJ , Kingsley PB , Taylor JS . WET, a T1‐ and B1‐insensitive water‐suppression method for in vivo localized 1H NMR spectroscopy. J Magn Reson B. 1994;104:1–10. 8025810 10.1006/jmrb.1994.1048

[14] Golay X , Petersen ET , Hui F . Pulsed star labeling of arterial regions (PULSAR): a robust regional perfusion technique for high field imaging. Magn Reson Med. 2005;53:15–21. 15690497 10.1002/mrm.20338

[15] Silver MS , Joseph RI , Hoult DI . Selective spin inversion in nuclear magnetic resonance and coherent optics through an exact solution of the Bloch‐Riccati equation. Phys Rev A Gen Phys. 1985;31:2753–2755. 9895827 10.1103/physreva.31.2753

[16] Lu H , Nagae‐Poetscher LM , Golay X , Lin D . Pomper M, van Zijl PC. Routine clinical brain MRI sequences for use at 3.0 Tesla. J Magn Reson Imaging. 2005;22:13–22. 15971174 10.1002/jmri.20356

