# Supplementary material for: A framework for motion correction of background suppressed arterial spin labeling perfusion images acquired with simultaneous multi‐slice EPI
Source: Magn Reson Med. 2018 Oct 12;81(3):1553–65. doi: 10.1002/mrm.27499 (PMC6492089; doi:10.1002/mrm.27499)
Supplement: Supplementary file 1 — FIGURE S1 Estimated motion parameters of all scans [file MRM-81-1553-s001.docx]

**Supporting information**

**Supporting Information Figure S1**


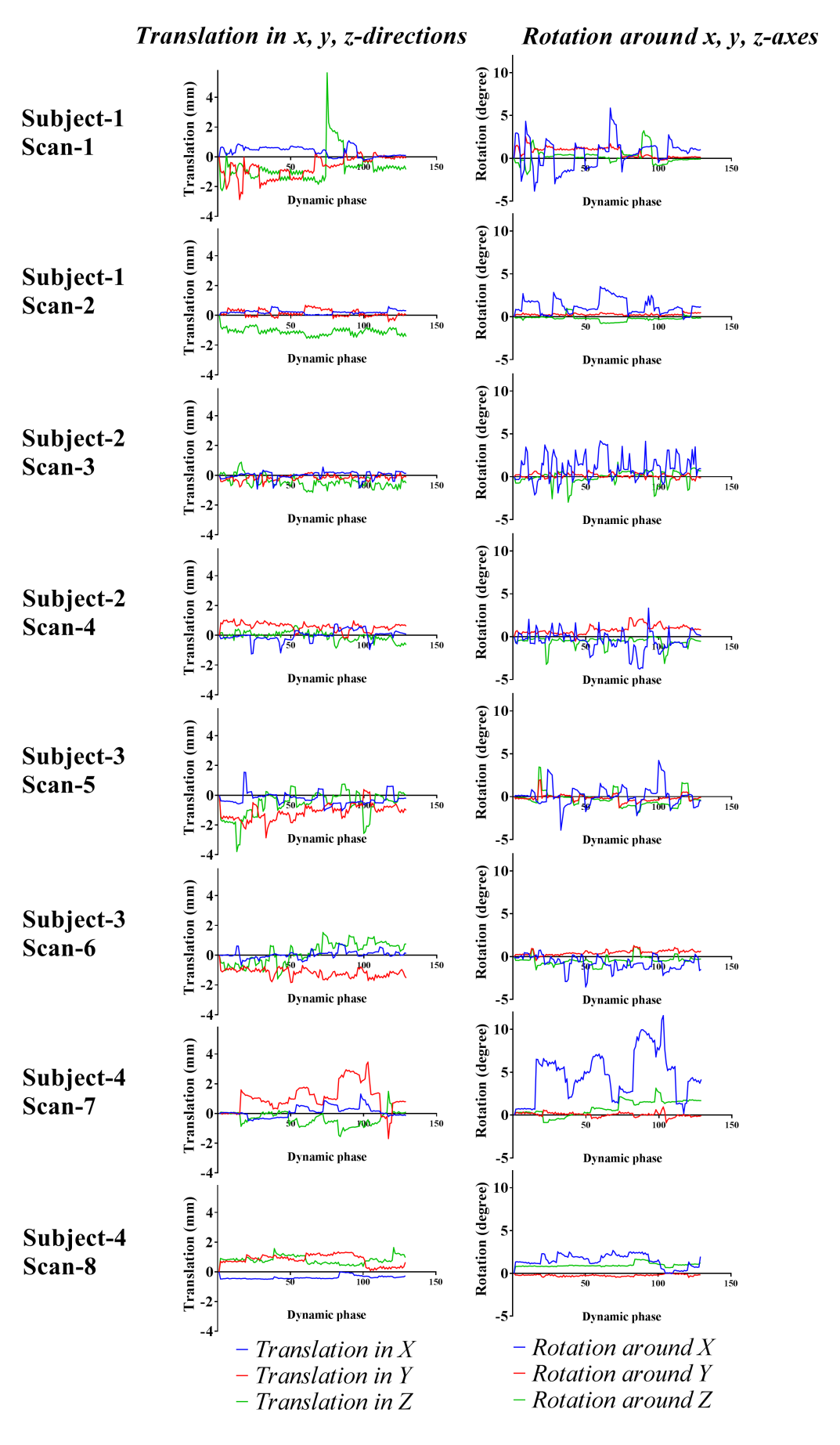


Supporting Information Figure S1: Estimated motion parameters of all scans.
